# Frequency and Causes of Traumatic and Non-traumatic Spinal Injury Reported at Two Major Tertiary Care Hospitals of Khyber Pakhtunkhwa

**DOI:** 10.7759/cureus.33230

**Published:** 2023-01-01

**Authors:** Sadiq Ullah, Syed Yousaf Khalid, Syed Muhammad Shoaib Iftikhar

**Affiliations:** 1 Neurosurgery, Rehman Medical Institute, Peshawar, PAK; 2 General Practice, Somerian Health Abu Dhabi, Abu Dhabi, ARE; 3 General Surgery, Letterkenny University Hospital, Letterkenny, IRL; 4 General Medicine, Rehman Medical Institute, Peshawar, PAK

**Keywords:** paraplegia, nervous system, trauma, intervertebral disc, lumbar vertebrae, spine, spinal injury

## Abstract

Introduction

Spinal cord injury, due to traumatic or non-traumatic causes, is a medically challenging and life-disrupting condition. The injury disrupts neural signaling and is a medical emergency requiring immediate treatment that can reduce long-term effects like paralysis or partial disability of the body. It has costly consequences both for individuals and families because it causes not only physical disability but dependency on others. The main objectives of the study were to determine the frequency of spinal injuries, their nature, and their causes.

Materials and methods

A descriptive exploratory study was conducted in the neurosurgery wards of two major hospitals of Peshawar, Lady Reading Hospital and Hayatabad Medical Complex. Necessary permission was taken from the administration of both hospitals before starting data collection. The duration of the study was three months, from October to December 2014. A total of 768 patients were retrieved from the ward records for 2013, from which a 50% random sample was taken (384 patients) while incomplete patient records were excluded. The data were transferred and recorded on a pre-constructed proforma covering all the required variables of the study. Finally, the data were transferred to SPSS 15 (SPSS Inc., Chicago) for analysis of descriptive statistics. In addition, comparisons were done by gender, hospitals, types of injuries, and causes of injuries. The chi-square test was used to compare groups for significant differences in frequencies, keeping p ≤0.05 as significant.

Results

Major factors for spinal cord injury were traumatic and non-traumatic. This study revealed that out of the total patients, 35% faced trauma as a cause of spinal disorder out of which 42%, 29%, and 21% were sudden falls, road traffic accidents (RTAs), and weight lifting, respectively. While non-traumatic causes were 52% mostly due to congenital anomalies (24%), stenosis (23%), and tumor (12%). Levels most commonly involved were lumbar (42.3%) followed by patients involving multiple levels (32.52%), L5-S1 (20.87%), thoracic (2.42%), and cervical (1.92%).

Conclusions

The traumatic injury was the leading cause of spinal cord injury in the present study where RTAs and falls contributed the most. Congenital abnormalities and spinal cord stenosis were more frequent among non-traumatic spinal cord injuries. The surgical approach was the only way of management practiced for spinal cord injuries in both of the tertiary care hospitals.

## Introduction

Spinal cord injury (SCI) can result from damage to the vertebrae and the surrounding tissue as well as direct injury to the spinal cord itself. This damage may cause temporary or permanent changes to sensation, mobility, strength, and bodily functions below the level of injury [1]. Although well protected by the bones and vertebrae of the spinal column, the spinal cord can be damaged in many ways. It can be compressed by an accident, injured by firearms injury (FAI), sudden trauma, etc. SCIs are highly disabling and deadly injuries with their treatment and rehabilitation exerting a great financial burden on patients, their families, and the social healthcare system [2,3].

Every year, around the world, between 250,000 and 500,000 people suffer an SCI, according to World Health Organization (WHO) 2013. In low-income countries, the mortality rate among people with SCI is higher than in developed countries [4]. Depending on the healthcare system's capacity, people with SCI have a 2-5 times higher risk of dying prematurely than those without SCI [5]. The high cost of health care in low-income nations is one of the main obstacles limiting the quality of life for those with SCI [6]. Additionally, 20-30% of SCI sufferers showed clinical signs of depression [7]. A recent estimate revealed that the annual incidence of SCI in the United States is approximately 54 cases per one million people, or approximately 17,810 new SCI cases each year, given the country's current population size of 329 million people. Those who pass away at the scene of the incident that resulted in the SCI are not counted as new SCI cases [8].

This retrospective descriptive study aimed to determine the types of SCIs in two major tertiary care hospitals in Peshawar, Khyber Pakhtunkhwa, Pakistan; additional objectives were to document the determinants of SCIs and their distribution by gender.

## Materials and methods

This is a descriptive study conducted at the Department of Neurosurgery, Lady Reading Hospital (LRH) and Hayatabad Medical Complex (HMC), Peshawar. The duration of the study was three months after approval from 1st October 2014 to 31st December 2014. The total figures for SCI patients from 2010 to 2013 in both hospitals were obtained from the registers and full data for the year 2013 (768 cases). The sample size of 384 out of 768 was calculated through computer-generated random sampling. The study population was patients admitted and operated on for spinal cord illness during the year 2013 in both hospitals. All the operated cases of SCI of the year 2013 in both hospitals of either gender were included. The exclusion criteria include patients with incomplete records.

Permission from the institutional ethical review committee was taken from both hospitals before conducting the study. Data were recorded on a pre-constructed proforma covering all the required variables of the study. Data were analyzed on SPSS version 15 (SPSS Inc., Chicago) for descriptive statistics and comparison with different variables. The chi-square test was applied and a p-value of ≤0.05 was considered significant.

## Results

Table 1 shows that out of 384 cases studied in two major hospitals of Peshawar, 311 (81%) were from LRH and 73 (19%) from HMC. Gender distribution was 212 (55%) males and 172 (45%) females. Age distribution showed 288 (75%) cases having ages above 20 years; however, 46 (12%) cases were below one year of age.

**Table 1:** Distribution of demographic data of patients by hospitals (n = 384) HMC, Hayatabad Medical Complex; LRH, Lady Reading Hospital.

Demographic data		HMC	LRH	Total f (%)
Gender	Male	38	174	212 (55.2)
	Female	35	137	172 (44.8)
Age groups (years)	<1	9	37	46 (12.0)
	1-10	2	11	13 (03.4)
	11-20	9	28	37 (09.6)
	21-30	24	56	80 (20.8)
	31-40	14	65	79 (20.6)
	41-50	7	58	65 (16.9)
	>50	8	56	64 (16.7)

Data for documented causes of SCI were available for 336 (87.50%) cases, with 48 cases missing, which were excluded. Table 2 shows the distribution of patients by hospitals based on gender and causes of traumatic injuries. Out of 336 cases, 134 (39.9%) patients suffered traumatic SCIs; among them, 56 (41.8%) were fall cases, 39 (29%) were road traffic accidents (RTAs), 28 (8.3%) due to weight lifting while 11 (3.3%) were because of FAIs. Significant differences were not noted for both gender and type of hospitals.

**Table 2:** Causes of traumatic spinal cord injuries in subjects by gender and hospital (n = 134) HMC, Hayatabad Medical Complex; LRH, Lady Reading Hospital.

Traumatic causes	Male f (%)		Total f (%)	p-Value	Female f (%)		Total f (%)	p-Value
	HMC	LRH		0.96	HMC	LRH		0.50
Firearm Injury	1	6	7		0	4	4	
Fall	7	31	38		3	15	18	
Weight lifting	4	15	19		3	6	9	
Road traffic accident	4	22	26		2	11	13	
Total	16 (11.94)	74 (55.22)	90 (67.91)		8 (5.97)	36 (26.86)	44 (32.83)	

Table 3 shows the data of 202 (60.5%) patients who had non-traumatic causes of SCIs, of which 49 (24%) were congenital type, 47 (23%) had stenosis, and 44 (22%) had other varied causes like tuberculosis (TB). The gender distribution of the non-traumatic patients showed that 49 subjects suffering from congenital injuries had a slight female preponderance (male = 21 and female = 28). Patients with stenosis (male = 23 and female = 24) showed no gender predilection, similar to patients with prolapsed intervertebral disc disease (male = 12, female = 12), and various syndromes (male = 6, female = 7). However, cases of tumors showed a marked female predisposition, with 9 (36%) males and 16 (64%) females being affected. Other causes, including TB, showed a male predilection (male = 29, female = 15). None of the gender differences reached statistical significance; however, the distribution of non-traumatic injuries by gender and hospitals was significant (p = 0.006 for males and p = 0.001 for females) as shown in Table 3.

**Table 3:** Causes of non-traumatic spinal cord injuries in subjects by gender and hospital (n = 202) PIVD, prolapsed intervertebral disc; HMC, Hayatabad Medical Complex; LRH, Lady Reading Hospital.

Non-traumatic causes	Male f (%)		Total f (%)	p-Value	Female f (%)		Total f (%)	p-Value
	HMC	LRH		0.006	HMC	LRH		0.001
Congenital	3	18	21		8	20	28	
Tumor	0	9	9		2	14	16	
PIVD	7	5	12		8	4	12	
Stenosis	3	20	23		2	22	24	
Syndrome	0	6	6		4	3	7	
Others	6	23	29		2	13	15	
Total	19 (9.4)	81 (40.1)	100 (49.5)		26 (12.8)	76 (37.6)	102 (50.5)	

**Figure 1:**
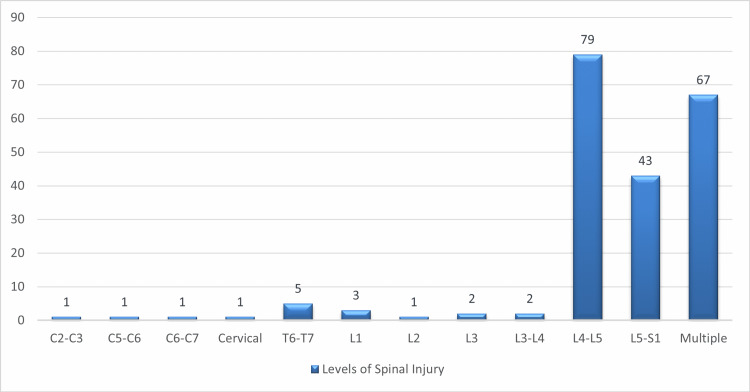
Levels of spinal injuries in subjects (n = 206)

Figure 1 shows the level of spinal injuries in 206 cases in which 79 subjects were having SCI levels of L4-L5, and 43 cases were having an injury of level L5-S1 and 67 cases were having multiple levels of injuries; some of the subjects (n = 5) also reported T6-T7 levels.

**Figure 2:**
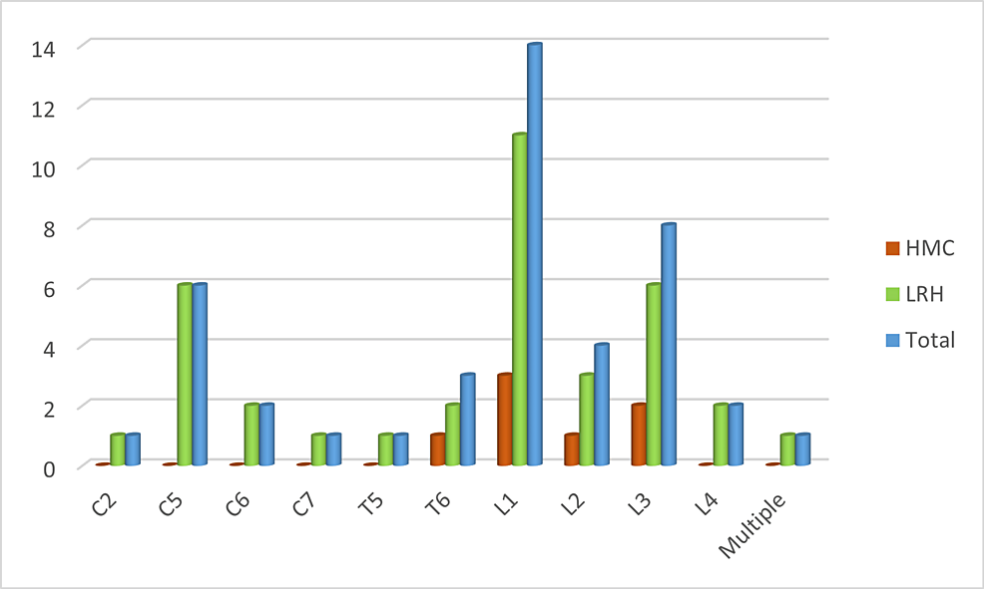
Vertebrae involved HMC, Hayatabad Medical Complex; LRH, Lady Reading Hospital.

Figure 2 depicts 43 (32% of all traumatic injuries) vertebral fractures, including 14 (32.5%) L1 fractures and 8 (18.6%) L3 fractures.

**Figure 3:**
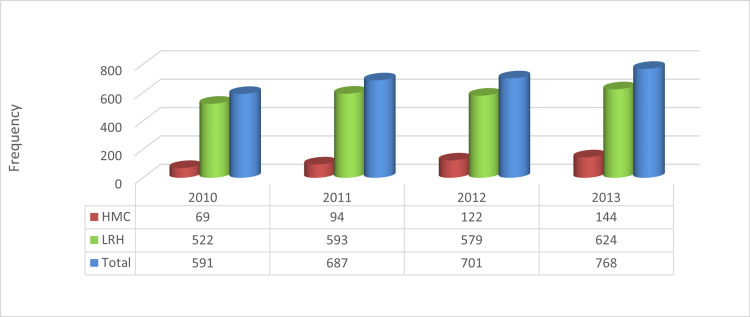
Trend of spinal injuries from 2010 to 2013 in selected hospitals (n = 768) HMC, Hayatabad Medical Complex; LRH, Lady Reading Hospital.

Figure 3 shows the trend of SCIs from 2010 to 2013, in which the increase in the number of patients having SCIs has been observed, i.e. a 29.94% increment in both hospitals. 

## Discussion

The term "spinal cord injury" refers to damage to the spinal cord due to trauma, disease, or degeneration. There is no definitive estimate of worldwide prevalence, but the estimated annual global incidence is 40-80 cases per million population. Up to 90% of these cases are due to traumatic causes, though the proportion of non-traumatic SCI appears to be growing. Avertible causes such as RTAs, falls, or violence cause most SCIs [4].

SCI may make a person dependent on caretakers and assistive technology is required usually to facilitate mobility, communication, self-care, or domestic activities. Clinically significant signs of depression are estimated to be seen in 20-30% of people with SCI, which in turn has a negative impact on improvements in functioning and overall health [4]. SCI has costly consequences for both individuals and society and the costs are higher than for other comparable conditions. The annual global incidence of traumatic SCI according to the Global Burden of Disease Study was estimated to be 0.93 million in 2016, with an age-standardized incidence rate of 13 (11-16) per 100,000 population [9]. The national costs because of hospitalizations related to SCI were estimated to be $1.7 billion in the United States [10].

According to a WHO report, males are at higher risk than females [4]. A study done in India shows that males were found to be more prone to SCI, which is similar to the findings in other studies as they are more engaged in outdoor work and hence are more prone to the spinal cord and/or other trauma [11]. In the present study, males suffered more from SCI than females.

Frequent causes of SCI are traumatic (fall, FAI, RTA, weight lifting) and non-traumatic (congenital, stenosis, tumors, etc.) [12]. In the current study, causes were divided into traumatic and non-traumatic. In non-traumatic causes, congenital is the leading cause followed by stenosis, while in traumatic causes fall is the leading cause of SCI followed by RTAs. RTAs are a frequent cause of traumatic spine injuries but they have become less common as a result of advancements in safety gear [13]. In this study, RTA was responsible for 29% of cases.

Studies done in India also show that falls (44.5%; 48.33%) were the leading cause of traumatic SCI followed by RTA (34.7%; 43.33%) [14,15]. Another study done by Chiu W [16] shows that falls are the leading cause of SCI in developing countries; however, RTA is the leading cause of SCI in developed countries. The present study also shows that falls are the leading cause of SCI and are responsible for 41.8% of cases.

Some people with SCIs have vertebral fractures while others have slipped discs. The study revealed that some patients with SCI also have vertebral fractures in which the L1 vertebra is involved in maximum patients. The same study done in India shows that the maximum vertebral fractures involved were L1 in paraplegic and fifth cervical in quadriplegic [14]. 

Another retrospective study conducted at the Royal Jordanian Rehabilitation Center (RJRC) revealed that the mean age of patients with SCI was 33 years, whereas 31 and 35 years of age for males and females, respectively [17]. The above-stated results of RJRC support the findings of our study regarding the SCI age group 20-40 years of age. 

The incidence and prevalence of SCI is still unknown in Pakistan. We believe studies like these should be conducted in different hospitals in the country to find out the disease burden in Pakistan.

## Conclusions

The traumatic injury was the leading cause of SCI in the present study where RTA and falls contributed the major chunk. Congenital abnormalities and spinal cord stenosis were more frequent among non-traumatic SCIs. The surgical approach was the only way of management practiced for SCIs in both of the tertiary care hospitals. 

Recommendation 

There is a strong need for public awareness through printed and electronic media regarding prevention from trauma like sudden falls, RTA, and weight lifting. The hospital must prioritize the evaluation and treatment of both traumatic and non-traumatic injuries. 
